# Editorial for the Special Issue on Tactile Sensing for Soft Robotics and Wearables

**DOI:** 10.3390/mi9120676

**Published:** 2018-12-19

**Authors:** Lucia Beccai, Massimo Totaro

**Affiliations:** Center for Micro-Biorobotics, Istituto Italiano di Tecnologia, Viale Rinaldo Piaggio 34, 56025 Pontedera (PI), Italy

Tactile feedback is needed for the interaction of humans with a worn device, and to enable robots to gather environmental cues and react to their surroundings. Tactile sensing has historically been a very active field of research, particularly for robotic systems (i.e., humanoids, robotic hands, and manipulators, etc.), and sensing elements were built in the past by mainly exploiting silicon-related technologies (i.e., MEMS, CMOS compatible devices) that relied on the fact that both the hosting system and the sensing components were made of rigid or low deformable materials. In this way, precise modeling and controllable fabrication processes are possible and can also be coupled with integrated conditioning and read-out circuitry, thus obtaining highly sensitive and reliable components and systems. The rise in wearable systems and, more recently, of soft robots, has imposed the need for flexible, stretchable, and conformable components to be mechanically coupled with a soft and highly deformable substrate (i.e., human tissues or a soft robot body). These requirements have introduced several new challenges, especially in revealing and distinguishing different mechanical cues, since both external stimulations and internal movements of the soft body cause similar deformations at the sensor level. Therefore, multidisciplinary efforts are mandatory and knowledge from different fields needs to be merged, e.g., from mechanical and electronic engineering, materials science, robotics, physiology and biomedical engineering, and computer science. Several challenging aspects must be tackled including the modeling of highly non-linear materials subjected to high deformations; the fabrication and integration of novel materials and functional structures; the design and fabrication of flexible and stretchable active components and hardware systems with proper signal processing and computational capabilities; the development of novel algorithms; and the definition of reliable protocols for the validation and testing of soft sensing systems. 

This Special Issue focuses on the emerging challenges in tactile sensing, both at a component and system level, for embedding mechanical sensing modalities in soft robots and wearable systems. 

One review [1] and four research articles [2,3,4,5] have been published in this Special Issue. These cover several topics in the field, spanning the recent technological advancements and the analysis of novel devices to the development of sensor network systems and improved algorithms as well as their clinical application on blind patients. 

In particular, Park et al. [1] reviewed the recent advancements in tactile sensing technologies. In their work, they addressed three main aspects: basic physiology in human tactile sensing, the requirements for the implementation of reliable tactile sensors, and the overview of novel materials and compounds for tactile devices. The present limitations in the field are discussed. For instance, according to the authors, there is still a relevant gap present between human and electronic skin. Human skin has a spatial resolution in the millimeter range, and is capable of simultaneously sensing different mechanical stimuli. To achieve a similar performance, several technological aspects need to be overcome: e-skins should be wearable and chemical-proof, stretchable and conformable, and integrate signal processing and transmission electronics. Finally, the authors discussed the long-term perspectives. From an economical point of view, currently, the technology is expensive and highly customized, limiting the high-volume manufacturing needed to reach an entry-market level. Hence, the authors concluded that particular effort in innovating mass production techniques and in discovering novel manufacturing procedures are needed.

In [2], Wu et al. proposed a flexible annular sectorial sensor that could be wrapped on the curved surface of a robotic arm that had a five layer structure and was designed for contact detection during robot movements. The transduction principle was based on a constant electric field on the upper and lower conductive layers. A mathematical model was developed to establish a relationship between the coordinates of the contact position and the corresponding electric field. The finite element method (FEM), using COMSOL® software, was used to simulate the sensor behavior. Results showed very good agreement between the experiments and theoretical and numerical predictions, indicating that the sensor performed well when wrapped around a robot arm.

From the electronic hardware point of view, Yamakawa et al. [3] presented a new network system with a high sampling rate based on the synchronization of a PC clock and data acquisition system. The high frequency (over 500 Hz) enabled the acquisition of data obtained from a large number of sensors. The scheme was made of multiple sensor nodes including PCs that were connected via the Ethernet for data communication and clock synchronization. The PC’s local clocks controlled the acquisition in each sensor node, while all clocks were globally synchronized over the network simultaneously to the data acquisition. Three different systems were implemented using this method: a high-speed tactile sensor node with a high-speed vision node, a high-speed tactile sensor node with three acceleration sensor nodes, and a high-speed tactile sensor node with two acceleration sensor nodes and a gyro sensor node. In all cases, the experiments showed that the timing error was less than 15 µs, well below the acquisition time of 2 ms. The results also confirmed that the proposed method could be applied in several areas such as robot control with real-time sensory feedback and intelligent transport systems as well as security and surveillance.

Machine learning is becoming a useful and powerful tool for tactile sensing in soft systems. Song et al. [4] faced the challenge of decoupling single components of three-axial force sensors by means of a machine learning method based on the improved back-propagation (BP) algorithm. This was applied in a 6 x 6 tactile sensor array to obtain the three-dimensional forces from the resistances of force-sensitive conductive pillars. The decoupling results demonstrated that the k-cross validation (k-CV) algorithm was an effective method to improve the decoupling precision of force components for the novel tactile sensor. The results were quite good, and in future studies, the authors plan to work on the recognition of the contact patterns due to three-dimensional forces, and on the development of different scales and densities for optimized performances.

Regarding the applications of haptic technologies in the biomedical field, this Special Issue includes a research paper on experiments with visual impaired (VI) people using a pin array matrix (PAM) for an orientation and mobility task. Brayda et al. [5] tested the effectiveness of a PAM representing the scaled map of a real room with the position of some target objects. After the participants haptically explored the PAM, they entered the real room to try to reach the targets three times. The first group of patients after each trial touched the same PAM again, while the second group could use an updated PAM that also included the positions that they previously reached in the room. This second group experienced significant improvements across trials, thus reducing both errors and completion time. As a result, the authors found that updated tactile feedback on programmable displays could be a powerful tool, giving much better performances with respect to conventional static tactile maps, therefore promoting more independent living for VI people.

We would like to take this opportunity to thank all of the authors for their efforts in presenting their research work in this Special Issue. We also thank all the reviewers for dedicating their time to assist in improving the quality of the submitted papers. Finally, we highly appreciate the remarkable support from the editorial staff of *Micromachines*.
